# Exploring endothelial dysfunction in major rheumatic diseases: current trends and future directions

**DOI:** 10.1007/s00109-025-02539-8

**Published:** 2025-04-14

**Authors:** Arshiya S. Anwar Husaini, Aseela Fathima, Dunia Halawa, Nada Aakel, Gian Luca Erre, Roberta Giordo, Hatem Zayed, Gianfranco Pintus

**Affiliations:** 1 Department of Biomedical Sciences, College of Health Sciences, QU Health, Qatar University, Doha, 2713 Qatar; 2 Rheumatology Unit, University Hospital (AOU) of Sassari, Sassari, Italy; 3 Department of Medicine, Surgery and Pharmacy, University of Sassari, Sassari, Italy; 4 Department of Biomedical Sciences, University of Sassari, Sassari, 07100 Italy; 5 Department of Medical Laboratory Sciences, College of Health Sciences, Sharjah Institute for Medical Research, University of Sharjah, Sharjah, 27272 United Arab Emirates

**Keywords:** Rheumatic diseases, Endothelial dysfunction, Chronic inflammation, Cardiovascular diseases, Immune system dysregulation

## Abstract

The relationship between rheumatic diseases (RDs) and endothelial dysfunction (ED) is intricate and multifaceted, with chronic inflammation and immune system dysregulation playing key roles. RDs, including Osteoarthritis (OA), Rheumatoid arthritis (RA), Systemic Lupus erythematosus (SLE), Ankylosing spondylitis (AS), Psoriatic arthritis (PsA), Sjogren’s syndrome (SS), Systemic sclerosis (SSc), Polymyalgia rheumatica (PMR) are characterized by chronic inflammation and immune dysregulation, leading to ED. ED is marked by reduced nitric oxide (NO) production, increased oxidative stress, and heightened pro-inflammatory and prothrombotic activities, which are crucial in the development of cardiovascular disease (CVD) and systemic inflammation. This association persists even in RD patients without conventional cardiovascular risk factors, suggesting a direct impact of RD-related inflammation on endothelial function. Studies also show that ED significantly contributes to atherosclerosis, thereby elevating cardiovascular risk in RD patients. This review synthesizes the molecular mechanisms connecting major RDs and ED, highlighting potential biomarkers and therapeutic targets. Ultimately, the review aims to enhance understanding of the complex interactions leading to ED in rheumatic patients and inform strategies to mitigate cardiovascular risks and improve patient outcomes.

## Introduction

Rheumatic diseases (RDs) represent a heterogeneous group of disorders marked by inflammation, pain, and structural damage affecting the joints, muscles, and connective tissues. They include autoimmune, autoinflammatory, and degenerative or metabolic conditions, and often present diagnostic challenges due to overlapping clinical manifestations and laboratory findings [1]. Among them, osteoarthritis (OA) is the most prevalent, primarily involving weight-bearing joints such as the knees, hips, and hands. Rheumatoid arthritis (RA) is a chronic inflammatory disease that typically targets the small joints and can lead to significant functional impairment. Systemic lupus erythematosus (SLE) is a complex autoimmune disorder with multisystem involvement. Ankylosing spondylitis (AS) predominantly affects the spine and sacroiliac joints, while psoriatic arthritis (PsA) manifests as inflammatory arthritis in individuals with psoriasis. Sjögren’s syndrome (SS) primarily targets the exocrine glands, resulting in dryness of the eyes and mouth. Systemic sclerosis (SSc) is a chronic autoimmune disease characterized by fibrosis of the skin and internal organs, accompanied by widespread vascular dysfunction. Lastly, polymyalgia rheumatica (PMR) is an inflammatory condition typically seen in older adults, presenting with pain and stiffness in the shoulders, neck, and hips, along with elevated inflammatory markers.

Endothelial dysfunction (ED) involves reduced nitric oxide (NO) production, increased oxidative stress, and enhanced pro-inflammatory and prothrombotic responses [2]. It is a hallmark of cardiovascular disease (CVD) and systemic inflammation. A growing body of evidence suggests that individuals with RDs are at increased risk of developing ED, primarily due to persistent inflammation and immune system dysregulation [3, 4]. Conversely, ED may exacerbate RDs by promoting further inflammation and tissue damage. This bidirectional relationship plays a critical role in the development of atherosclerosis and significantly contributes to cardiovascular risk in RD patients—even in the absence of traditional risk factors. These insights highlight the potential for RD-related inflammation to directly impair endothelial function [3, 4], reinforcing the need for a comprehensive understanding of the underlying mechanisms linking RDs and ED.

This review aims to explore recent literature investigating the link between major rheumatic diseases, including OA, RA, SLE, AS, PsA, SS, SSc, PMR and ED. By delineating the distinct pathophysiological pathways involved, the review seeks to identify potential biomarkers, therapeutic targets, and preventive strategies to mitigate cardiovascular risk in RD patients. Ultimately, this synthesis aspires to inform clinical management and guide future research directions.

## Search methodology

The literature search was conducted using a variety of online databases, such as PubMed and Science Direct, ProQuest, and DOAJ. To ensure the identification of relevant studies, the word “Endothelial dysfunction” was used along with different keywords, including “Osteoarthritis, Rheumatoid arthritis, systemic lupus erythematosus, ankylosing spondylitis, psoriatic arthritis, Sjogren’s, scleroderma, and polymyalgia”. Articles were screened and selected, with a restriction date on publication dates from 10 years. However, there is no restriction on the experimental subjects/models used, the mode and duration, or other particular experimental details.

## Rheumatoid arthritis and osteoarthritis and endothelial dysfunction

RA is a chronic autoimmune disease characterized by systemic inflammation that primarily targets the synovial joints, resulting in progressive disability and increased mortality [5]. Affecting approximately 1% of the global population, RA typically manifests between the ages of 50 and 60 and is 2–3 times more prevalent in females [6, 7]. Although its exact etiology remains incompletely understood, a combination of genetic predisposition and environmental triggers is thought to drive disease onset [6, 7]. RA is associated with a twofold increased risk of myocardial infarction and a 50% rise in CVD-related mortality, largely due to chronic immune activation, endothelial injury, and dysfunction [8–10].

In contrast, OA, the most prevalent chronic joint disease, is marked by cartilage degradation, synovial inflammation, and subchondral bone remodeling [11]. Traditionally considered a non-inflammatory, degenerative condition, growing evidence suggests that OA may have systemic vascular implications mediated through ED. A bidirectional relationship between OA and ED has been proposed, with studies linking hand OA to increased atherosclerosis and impaired endothelial markers, potentially through mechanisms involving inflammation, oxidative stress, and diminished joint perfusion [11]. Synovial vascular pathology in late-stage knee OA, characterized by biomechanical stress-induced angiogenesis and elevated angiogenic markers (e.g., VEGF, hypoxia-inducible factors), further implicates endothelial stress in OA progression [12].

The molecular mechanisms underlying the increased risk of atherosclerosis RA are not yet fully understood. However, chronic inflammation and immune dysregulation are known to directly contribute to ED, ultimately leading to vascular stiffening, plaque formation, and elevated CVD risk (Table 1) [13]. RA patients also tend to have elevated CIMT, reflecting accelerated arterial plaque formation associated with chronic inflammation [14–16]. In this context, circulating factors in RA patient sera have been shown to induce oxidative stress, as well as pro-angiogenic and profibrotic changes in endothelial cells, thereby exacerbating vascular dysfunction [17].

**Table 1:** Molecular pathways and therapeutic targets in rheumatoid arthritis-associated endothelial dysfunction

Parameters involved in RA	Pathway(s)/mechanism involved	Potential treatment/diagnostic tool
TNF- α	Contributes to NO deficiency via upregulated adhesion molecules (VCAM- 1, ICAM- 1), impaired vasodilation, increased oxidative stress, and decreased NO availability [8]	Anti-TNF-α therapy reduces circulating ED biomarkers, including sVCAM- 1, MCP- 1, ADMA, and plasma heparan sulfate/heparin (HS/H) levels [8]
	Elevates oxLDL levels, activating the LOX- 1/NFκB/Arg2 signaling pathway [18]	Anti-TNF-α therapy improves articular symptoms and endothelial function by reducing LOX- 1, vascular oxLDL, and Arg2 levels, potentially conferring cardiovascular protection [18]
NO System	Elevated NO₂⁻ and NO₃⁻ levels in younger and middle-aged RA patients with CHD correlate with milder ED and shorter disease duration; older patients exhibit lower NO₂⁻ levels and more severe ED. VEGF levels rise with longer RA duration [19]	
	Reduced expression of the − 786 T > C eNOS promoter variant correlates with increased ED risk [20]	
	RA promotes cerebrovascular ED via imbalance in the arginase/NOS pathway [21]	Arginase inhibition represents a promising therapeutic strategy, alongside conventional anti-rheumatic treatments, to reduce CVD risk in RA patients [21]
	Dimethylarginines, acting as NOS inhibitors, may contribute to RA-associated atherosclerosis through gene variants of AGTX- 2. [22]	Dimethylarginines serve as novel, independent biomarkers of CVD in conditions characterized by vascular complications [23, 24]
	Interplay between endothelial COX- 2 and NOS pathways [25]	NO-releasing NSAIDs as therapeutic options; plasma IL- 1β, TNF-α, and MIP- 1α as potential diagnostic biomarkers of ED in RA [25]
Microvascular ED	High peripheral microvascular ED in RA patients accelerates atherosclerosis, significantly increasing future cardiovascular event risk [5]	
IMA	Elevated serum IMA levels in RA patients are associated with inflammation, oxidative stress, and increased CIMT [26]	
EPCs	Adiponectin enhances EPC migration and tube formation via VEGF activation through the MEK/ERK pathway; impaired NO/eNOS signaling disrupts EPC mobilization, resulting in reduced EPC levels in RA patients with ED [10]	Inhibition of adiponectin reduces joint swelling, bone destruction, and angiogenic marker expression in collagen-induced arthritis models Therapies aimed at suppressing inflammation and stimulating EPCs may prevent and manage early ED and accelerated atherosclerosis in RA patient [27]
BDNF	Alterations in the BDNF signaling pathway and its receptor may link ED to cognitive impairment [28]	

Key molecular pathways and associated therapeutic/diagnostic tools in Rheumatoid Arthritis-related endothelial dysfunction. Parameters include cytokines, enzymes, and biomarkers linked to vascular pathology. Abbreviations: *TNF-α* tumor necrosis factor-alpha, *NO* nitric oxide, *oxLDL* oxidized *LDL, ADMA *asymmetric dimethylarginine,* VCAM-1* vascular cell adhesion molecule-1, *ICAM-1* intercellular adhesion molecule-1, *HS/H* heparan sulfate/heparin *VEGF* vascular endothelial growth factor, *NOS* nitric oxide synthase, *eNOS* endothelial NOS, *NSAIDs* nonsteroidal anti-inflammatory drugs, *IMA* ischemia-modified albumin, *CIMT* carotid intima-media thickness, *EPCs* endothelial progenitor cells, *BDNF* brain-derived neurotrophic factor, *CHD* Coronary Heart Disease

**Fig. 1:**
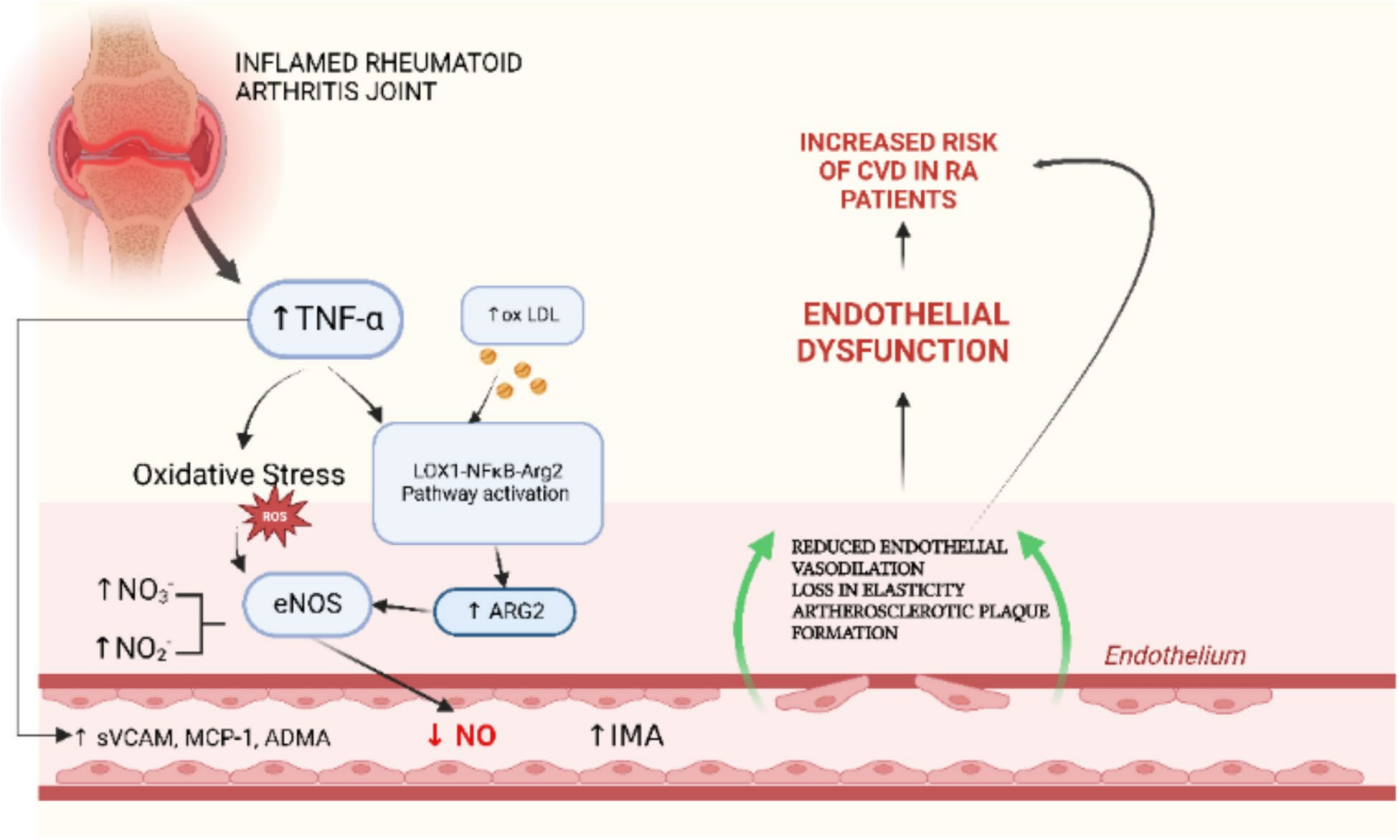
Mechanistic illustration of endothelial dysfunction in rheumatoid arthritis. Inflammatory cytokines, particularly TNF-α released from inflamed joints, promote oxidative stress and increase oxidized LDL (oxLDL). This activates the LOX1–NFκB–Arg2 signaling pathway, leading to ARG2 upregulation and eNOS uncoupling, resulting in decreased nitric oxide (NO) bioavailability. The reduction in NO impairs endothelial function, as indicated by increased soluble VCAM- 1, MCP- 1, ADMA, and intima-media thickness (IMT). These changes collectively contribute to reduced vasodilation, vascular stiffness, plaque formation, and a heightened cardiovascular disease risk in RA patients

RA is associated with increased hypertension prevalence, exacerbating cardiovascular risk due to chronic inflammation and ED. These elements likely form a mutually reinforcing triad, underscoring the importance of early ED identification [29]. Microvascular ED is also prevalent, affecting approximately one-third of RA patients without prior CVD, as evidenced by digital pulse amplitude tonometry [5]. Central to RA-related ED is the nitric oxide synthase/endothelial nitric oxide synthase (NOS/eNOS) pathway [18–21, 25, 30]. Biomarkers such as circulating dimethylarginines and nitrites hold promise for ED diagnosis and management in RA [30] (Table 1). Rodent arthritis models indicate that elevated NOS activity may transiently mitigate ED during early disease phases [25]. Thus, chronic oxidative stress, autoimmunity, and pro-inflammatory cytokines collectively accelerate atherosclerosis, increasing cardiovascular events and arrhythmias in RA patients. Figure 1 provides a visual summary of the molecular mechanisms by which inflammation, oxidative stress, and dysregulation of the NOS/eNOS pathway contribute to ED and the increased cardiovascular risk observed in RA.

Ischemia-modified albumin (IMA) represents another promising ED biomarker, with elevated IMA levels correlating with inflammation, oxidative stress, and increased CIMT in RA [26]. Reduced peripheral vasodilatory capacity, observed in approximately one-third of RA patients, aligns closely with elevated IMA levels and increased Disease Activity Score- 28 (DAS- 28) [26]. Despite the absence of flow-mediated dilation (FMD) measurements, a well-established parameter for ED evaluation, these findings emphasize IMA’s clinical relevance in RA [26].

Endothelial progenitor cells (EPCs), crucial for endothelial repair and atherosclerosis mitigation, are frequently depleted in RA. Reduced EPC levels correlate with bone erosion, advanced age, and decreased high-density lipoprotein (HDL) [31] Disrupted NO/eNOS signaling exacerbates ED by impairing EPC mobilization, while adiponectin enhances EPC function through VEGF-mediated pathways, highlighting potential therapeutic targets [10]. Anti-TNF therapy can restore EPC populations, and vitamin D deficiency, common in RA, diminishes EPC counts and correlates with greater disease activity, insulin resistance, and ED [31]. Studies on Indian RA patients have further linked impaired FMD and CIMT to inflammation (CRP, TNF-α), serum nitrite levels, DAS- 28 scores, and EPC depletion, suggesting therapeutic benefits from anti-inflammatory and EPC-enhancing treatments [27].

Oxidative stress, often assessed through malondialdehyde (MDA), contributes significantly to atherosclerosis and ED in RA [32]. Asymmetric dimethylarginine (ADMA), another established ED and CVD biomarker, is elevated in RA [24]. Elevated ADMA and urinary cartilage degradation products (uCTX-II) have also been detected in hypertensive individuals with OA compared to hypertensive controls without OA, implying that ED may further exacerbate joint deterioration in OA [33]. The association between ADMA and uCTX-II underscores the potential relationship between vascular dysfunction and joint pathology in OA, although precise mechanisms linking biomechanical stress, inflammation, and ED require further investigation.

RA additionally encompasses peripheral neuropathies, nerve lesions, multi-organ dysfunction, and cognitive impairment. Endothelial-derived brain-derived neurotrophic factor (BDNF) may link ED to neurological deficits, as disrupted BDNF signaling has been reported in these conditions [28]. Genetic associations also play a role; the G allele of rs646776 (1p13.3) is notably more frequent in RA patients and correlates with higher ED and CVD incidence [34].

In conclusion, RA involves multiple interconnected mechanisms, and despite significant advances, no definitive cure currently exists. Investigating RA through the perspective of ED may provide valuable insights, paving the way for novel interventions aimed at reducing vascular complications and enhancing patient outcomes.

## Systemic lupus erythematosus and endothelial dysfunction

Recent studies on RDs have increasingly emphasized the significance of ED in SLE. CVD, which is more prevalent and presents distinct features in SLE patients, is strongly associated with impaired FMD [35–37] and compromised endothelial integrity [38, 39]. Consistently, multiple studies have reported increased CIMT in SLE patients, indicative of subclinical atherosclerosis and highlighting early vascular damage in this condition [40, 41]. ED in SLE arises from several interrelated factors, including autoantibodies, proinflammatory cytokines, thrombotic microangiopathy, oxidative stress, and alterations in immune cell populations such as T cells and EPCs [38, 42, 43].

**Fig. 2:**
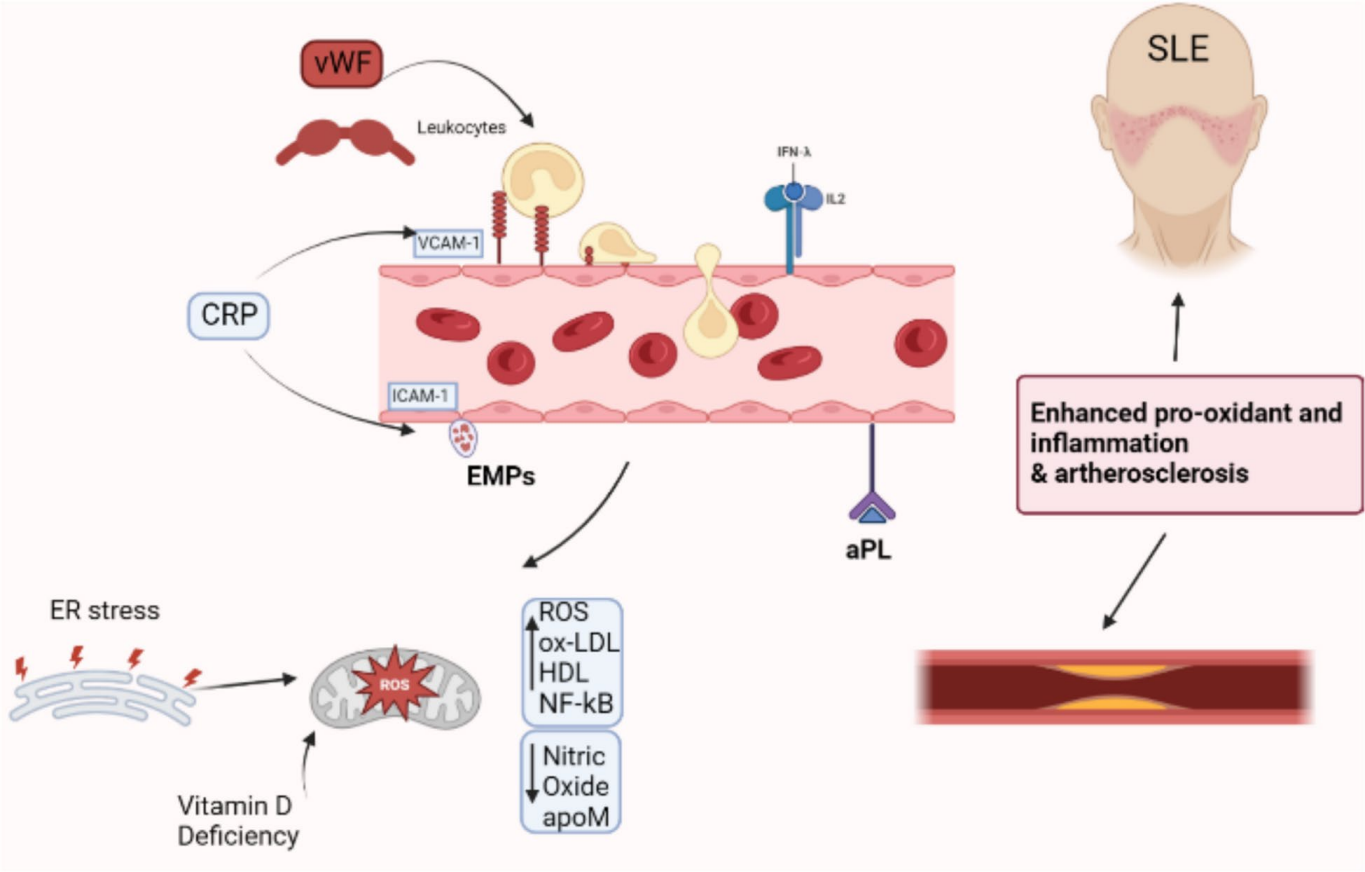
Systemic lupus erythematosus-induced endothelial dysfunction and atherosclerosis. Inflammatory mediators such as CRP, vWF, and leukocytes promote upregulation of adhesion molecules (VCAM- 1, ICAM- 1) and release of endothelial microparticles (EMPs). Type I interferons (IFN-α), interleukins (e.g., IL- 2), and antiphospholipid antibodies (aPL) further contribute to endothelial activation. Concurrently, vitamin D deficiency induces ER stress and reactive oxygen species (ROS) production, increasing oxidative stress (ox-LDL, NF-kB) and reducing nitric oxide (NO) and apoM, thereby impairing vascular homeostasis. These interconnected pathways promote inflammation, pro-oxidant states, and vascular damage, accelerating atherosclerosis in SLE

FMD-associated ED is characterized by increased oxidative stress, decreased NO bioavailability and increased endoplasmic reticulum (ER) stress [36, 44]. Excessive reactive oxygen species (ROS), predominantly generated by NADPH oxidase and uncoupled eNOS, decrease NO availability, impair vasodilation, and exacerbate endothelial damage (Fig. 2) [44, 45]. Plasma from SLE patients induces ROS production in human umbilical vein endothelial cells (HUVECs), a process attenuated by inhibiting ER stress and NADPH oxidase activity [44]. Since ER stress directly limits NO synthesis, targeting these pathways represents a promising strategy to mitigate CVD risk in SLE [44]. Key biomarkers and therapeutic strategies for SLE-associated ED are summarized in Table 2.

**Table 2:** Biomarkers of and their therapeutic relevance in systemic lupus erythematosus

Biomarkers	Mechanism of action in ED	Therapeutic relevance
CRP	Indicates systemic inflammation; promotes endothelial activation via adhesion molecule upregulation [46]	Statins and anti-inflammatory treatments reduce CRP, enhancing endothelial function [46]
sVCAM- 1	Facilitates leukocyte-endothelial adhesion; marker of endothelial activation [47]	HCQ reduces sVCAM- 1, attenuating endothelial activation [47]
ICAM- 1	Promotes leukocyte-endothelial adhesion; indicates endothelial activation [47]	HCQ lowers ICAM- 1, reducing endothelial activation [47]
vWF	Released upon endothelial injury, facilitating platelet adhesion [47]	Antioxidants and HCQ decrease vWF levels, reducing thrombosis risk [47, 48]
EMPs	Released from activated or apoptotic endothelial cells; reflect endothelial injury [49]	Immunosuppressive therapy reduces EMP release, improving endothelial health [49]
IMT	Indicator of arterial wall thickness and subclinical atherosclerosis [40, 41]	Statins interventions reduce IMT and slow atherosclerosis [50]
FMD	Reflects endothelial-dependent vasodilation and NO availability [35, 36, 37]	Vitamin D enhanced NO secretion by EPCs, improving FMD and endothelial function [51]
Pentraxin 3	An indicator of cutaneous disease activity [52]	Anti-inflammatory treatments decrease pentraxin 3, enhancing vascular health [53]
apoM	HDL component protective against endothelial damage and inflammation [54]	HDL-raising therapies can restore apoM levels and endothelial function [54]
IFNs	Trigger endothelial activation, platelet activation, and impaired endothelial function [55, 56]	Potential future therapies targeting IFN pathways could improve vascular health [55, 56]
Antiphospholipid Antibodies (aPL)	Promote thrombosis and endothelial activation (LA, aCL, aβ2GPI) [46]	Statins treatments reduce aPL-induced vascular damage [46]
CX3 CL1 (Fractalkine)	Enhances monocyte-endothelial adhesion, worsening endothelial activation [57]	Targeting the CX3 CL1-CX3 CR1 axis may reduce monocyte adhesion and improve endothelial function[57]

Key biomarkers and their mechanisms in SLE-related endothelial dysfunction, alongside potential therapeutic intervention. Abbreviations:*CRP* C-reactive protein, *sVCAM-1* soluble vascular cell adhesion molecule-1, *ICAM-1* intercellular adhesion molecule-1, *vWF* von Willebrand factor, *EMPs*: endothelial microparticles, *IMT* intima-media thickness, *FMD* flow-mediated dilation, *NO* nitric oxide, *apoM* apolipoprotein M, *HDL* high-density lipoprotein, *IFNs* interferons, *aPL* antiphospholipid antibodies, *LA* lupus anticoagulant, *aCL* anticardiolipin antibodies, *aβ2GPI* anti-β2 glycoprotein I antibodies, *HCQ* hydroxychloroquine

Immune dysregulation further intensifies endothelial damage, with autoantibodies, cytokines, and type I interferons (IFNs) driving endothelial activation. Elevated IFN levels impair FDM and accelerate atherosclerosis, while antiphospholipid antibodies (aPL) contribute to thrombosis and vascular injury [46, 56, 58]. Increased levels of endothelial activation markers such as soluble VCAM- 1 (sVCAM- 1), von Willebrand factor (vWF), and endothelial microparticles (EMPs) correlate closely with disease severity and vascular damage [46, 56]. Additionally, the CX3 CL1-CX3 CR1 signaling axis exacerbates ED by enhancing monocyte-endothelium adhesion [57].

Vitamin D deficiency exacerbates ED in SLE patients, correlating with heightened CVD risk and vascular stiffness [39]. Murine lupus models confirm that low vitamin D worsens ED and disrupts angiogenesis [39]. Moreover, vitamin D deficiency is linked to increased expression of type I interferon-stimulated genes (ISGs) in both humans and mice with lupus, accompanied by enhanced ROS production and ER stress within endothelial cells [39, 55]. These findings suggest that vitamin D supplementation may be beneficial for reducing CVD risk in SLE patients. Figure 2 illustrates the multifaceted mechanisms by which immune dysregulation and vitamin D deficiency contribute to ED and accelerated atherosclerosis in SLE, emphasizing the roles of type I interferons, adhesion molecules, oxidative and ER stress, and key biomarkers such as VCAM- 1, vWF, and EMPs.

Further research is needed to clarify the molecular mechanisms linking SLE-associated factors, ED biomarkers, and cardiovascular outcomes, thereby facilitating targeted therapeutic interventions. Potential therapeutic strategies include matrix metalloproteinase (MMP) inhibitors and vascular risk markers; however, rigorous clinical trials are necessary to validate their effectiveness[59].

Activation of peroxisome proliferator-activated receptor β/δ (PPARβ/δ) has shown promise in reversing NO impairment induced by SLE plasma through specific inhibition of endothelial ER stress, indicating therapeutic potential [44]. Nonetheless, additional studies are required to elucidate detailed molecular pathways and to confirm clinical efficacy of PPARβ/δ agonists.

Statins have emerged as potential agents for addressing ED in SLE due to their ability to maintain vascular function, reduce inflammation, and stabilize atherosclerotic plaques [46]. Similarly, despite mixed clinical results [60], vitamin D supplementation—particularly cholecalciferol—appears promising for reducing cardiovascular risk, promoting endothelial repair, and restoring myeloid angiogenic cell (MAC) function in SLE patients [39]. Future investigations should aim to identify optimal serum vitamin D concentrations and assess long-term cardiovascular outcomes.

Hydroxychloroquine (HCQ) has also demonstrated considerable therapeutic promise. In murine SLE models, HCQ reduces hypertension and preserves endothelial function by lowering oxidative stress and enhancing NO bioavailability [61]. Furthermore, HCQ improves not only general disease activity but also endothelial function directly, as indicated by decreased levels of soluble adhesion molecules correlating with higher HCQ blood concentrations [47]. Early HCQ administration in experimental models has notably enhanced endothelial function prior to the onset of overt renal pathology [48]. HCQ thus demonstrates significant potential in improving endothelial function and reducing hypertension in SLE by lowering oxidative stress, enhancing NO bioavailability, and attenuating endothelial activation [47, 48] (Table 2). These findings underscore HCQ’s dual role in disease control and vascular protection, highlighting the importance of early intervention and personalized dosing strategies.

## Ankylosing spondylitis and endothelial dysfunction

AS is a chronic inflammatory arthritis primarily affecting the spinal joints and ligaments, with predominant involvement of the lumbar region [62, 63]. Emerging evidence indicates that AS patients face an elevated risk of premature atherosclerosis, driven by systemic inflammation, vascular intimal hyperplasia, and adventitial fibrosis. These pathological changes, closely linked to immune dysregulation and chronic inflammation characteristic of immune-mediated disorders, accelerate atherosclerotic progression, thereby heightening CVD risk in this population [64].

A prospective case–control study compared AS patients to individuals with non-inflammatory rheumatic conditions, assessing lipid profiles and their correlation with inflammatory markers via carotid artery CIMT measurements. Atherosclerosis was detected in 34.29% of AS patients versus 5.71% of controls, alongside significantly reduced high-density lipoprotein cholesterol (HDLc) levels in AS cohorts—a key contributor to CVD risk [63, 64]. Vascular ultrasound further demonstrated structural vascular damage, with atheromatous plaques observed in 21.43% of AS patients compared to 4.29% of controls, underscoring the pronounced atherosclerosis burden in AS [64].

EPCs, bone marrow-derived stem cells essential for endothelial repair and atherosclerotic protection, are depleted in AS patients. EPC levels inversely correlate with disease duration, activity, and inflammatory markers, while positively associating with endothelial function measured via carotid intima-media thickness and CIMT [63]. A cross-sectional study revealed diminished EPC populations in AS patients independent of traditional CVD risk factors (e.g., smoking, hypertension), suggesting that intrinsic inflammatory pathways—elevated erythrocyte sedimentation rate (ESR), C-reactive protein (CRP), tumor necrosis factor-alpha (TNF-α), interleukin- 6 (IL- 6), and interleukin- 1 (IL- 1)—drive CVD risk independently [62].

**Fig. 3:**
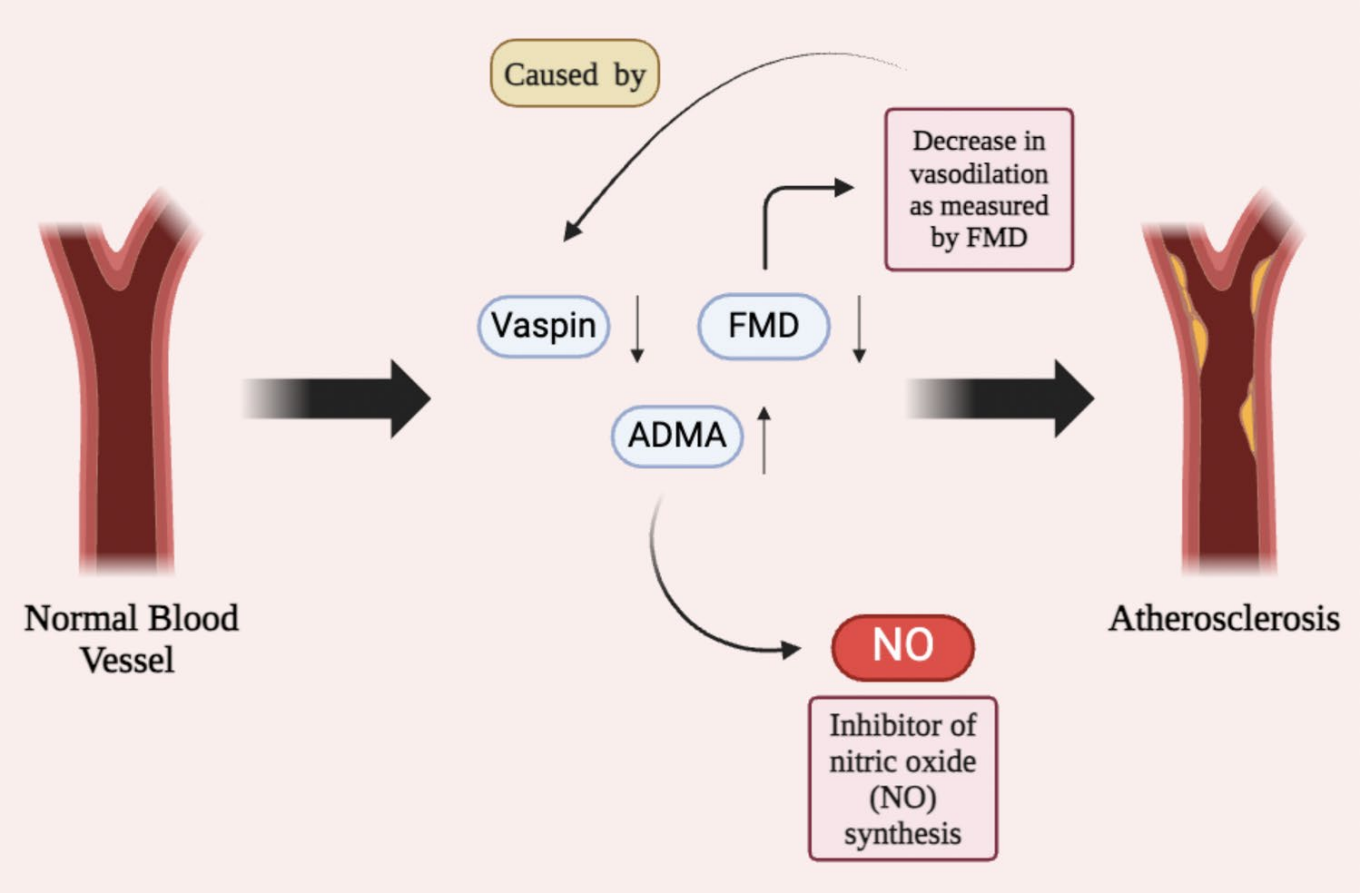
Endothelial dysfunction and atherosclerosis in ankylosing spondylitis. Reduced levels of vaspin lead to decreased flow-mediated dilation (FMD) and elevated concentrations of asymmetric dimethylarginine (ADMA), an endogenous inhibitor of nitric oxide (NO) synthesis. The resulting decrease in NO impairs vasodilation, contributing to endothelial dysfunction and the progression from a normal blood vessel to atherosclerosis

Vaspin, a visceral adipose tissue-derived adipokine with anti-inflammatory and insulin-sensitizing properties, has emerged as a novel biomarker. AS patients exhibit reduced serum vaspin levels, which correlate with impaired endothelial function (measured by FMD) and elevated pro-inflammatory cytokines, positioning vaspin as a potential early indicator of atherosclerosis [65]. Additional biomarkers implicated in AS-related atherosclerosis include elevated asymmetric dimethylarginine (ADMA), an endogenous NOS inhibitor, and persistent platelet activation post-anti-TNF therapy, evidenced by increased platelet-monocyte complexes (PMC) and soluble CD40 ligand (sCD40L) levels [63]. Figure 3 depicts the contribution of reduced vaspin levels and increased ADMA concentrations to impaired NOS and ED in AS, highlighting the progression from reduced vasodilation to atherosclerosis.

Therapeutic exploration of angiotensin II receptor blockers (ARBs), such as olmesartan, demonstrates their immunomodulatory potential, including cytokine suppression and improved physical function and quality of life (QOL) in AS patients [66]. However, clinical strategies for mitigating CVD risk in AS remain limited, necessitating comprehensive cardiovascular risk assessments integrating vaspin, endothelial function, and inflammatory biomarker monitoring.

## Psoriatic arthritis and endothelial dysfunction

PsA is a chronic inflammatory musculoskeletal disorder frequently associated with psoriasis, manifesting as axial or peripheral arthritis, enthesitis, dactylitis, or a combination of these phenotypes [2]. While 20–30% of plaque psoriasis patients develop PsA, 10–15% experience PsA onset prior to cutaneous symptoms [67]. Contrary to earlier perceptions of mild severity, PsA induces joint dysfunction and quality-of-life impairment comparable RA [2]. ED mechanistically links PsA to CVD, contributing to elevated cardiovascular morbidity and mortality, including heightened risks of myocardial infarction (MI) and stroke[2, 68].

A three-year longitudinal study of juvenile PsA (JPSA) patients revealed significantly impaired FMD of the brachial artery compared to healthy controls, independent of traditional CVD risk factors [69]. Reduced FMD correlated with disease duration, systemic inflammation, and functional disability, implicating early disease onset, heightened activity, and genetic predispositions (e.g., HLA DR5 and HLA DRw8 variants) in accelerating cardiovascular risk [69]. Similarly, adult PsA cohorts exhibit increased CIMT, diminished FMD, reduced endothelial progenitor cell (EPC) levels, and dyslipidemia—notably low high-density lipoprotein (HDL) cholesterol—consistent with accelerated atherosclerosis [70, 71].

Chronic inflammation in PsA is marked by elevated CRP, erythrocyte sedimentation rate (ESR), proinflammatory cytokines (IL- 6, TNF-α, IL- 1β), adipokines (leptin, adiponectin), and adhesion molecules (ICAM- 1, VCAM- 1), all implicated in ED and atherosclerotic progression [2, 67, 71–73]. Non-inflammatory pathways, including hypothalamic–pituitary–adrenal (HPA) axis dysregulation (elevated cortisol) and autonomic nervous system imbalances, exacerbate cardiovascular risk, particularly in PsA patients with comorbid depression [72, 74]. Females with PsA display higher depressive scores than males, likely due to biological, psychological, and social factors [72]. Platelet hyperactivity, evidenced by elevated P-selectin and platelet-derived microparticles in psoriasis/PsA patients, further amplifies thrombotic risk [71].

Circulating endothelial progenitor cells (CEPCs), bone marrow-derived cells critical for endothelial repair and vascular homeostasis, are reduced in PsA patients (CD133 +/KDR + and CD34 + subsets), impairing endothelial regeneration and exacerbating CVD risk [75, 76]. Additional risk factors include obesity, arterial stiffness, hypertension, diabetes, and dyslipidemia, which synergistically elevate major adverse cardiovascular event (MACE) incidence [71, 73]. Therapeutic agents targeting inflammatory pathways, such as TNF-α inhibitors, IL- 12/IL- 23 inhibitors (ustekinumab), and IL- 17/IL- 23 inhibitors, improve psoriasis management and may mitigate CVD risk. Observational studies report a 55% reduction in MI risk among TNF inhibitor-treated psoriasis patients compared to topical therapy cohorts [73, 77]. Disease-modifying antirheumatic drugs (DMARDs) and biologics targeting the IL- 23/Th17 axis attenuate subclinical atherosclerosis progression, evidenced by reduced fat attenuation indices in imaging studies [77]. However, further research is needed to clarify the cardiovascular benefits of IL- 17/IL- 23 inhibitors.

## Sjogren’s syndrome and endothelial dysfunction

SS is a chronic autoimmune disorder marked by lymphocytic infiltration of exocrine glands, primarily affecting lacrimal and salivary glands, resulting in sicca symptoms and glandular dysfunction [78, 79]. SS is classified as primary SS (pSS) when occurring in isolation and secondary SS when associated with other autoimmune conditions [80]. ED, a key pathophysiological feature in SS, exacerbates systemic disease activity and contributes to cardiovascular and neurological complications [81].

Patients with pSS exhibit distinct markers of endothelial damage. A recent study identified significantly reduced FMD, elevated inflammatory cytokines (IL- 6, TNF-α, β2-microglobulin), and higher titers of anti-SSA/SSB antibodies and EULAR Sjögren’s Syndrome Disease Activity Index (ESSDAI) scores, reflecting severe vascular inflammation and impaired vascular tone [79]. However, the limited demographic diversity of the cohort and the narrow focus on inflammatory mechanisms underscore the need for broader, more comprehensive investigations. Other studies have reported elevated levels of adropin, a peptide involved in modulating endothelial nitric oxide availability, angiogenesis, and resistance to oxidative stress, in patients with SS [80]. Notably, adropin levels showed a negative correlation with the SS Disease Damage Index (SSDDI) and a positive correlation with anti-SSA/Ro52 antibody titers, suggesting a compensatory role in counteracting endothelial damage during disease progression [80, 82–85].

**Fig. 4:**
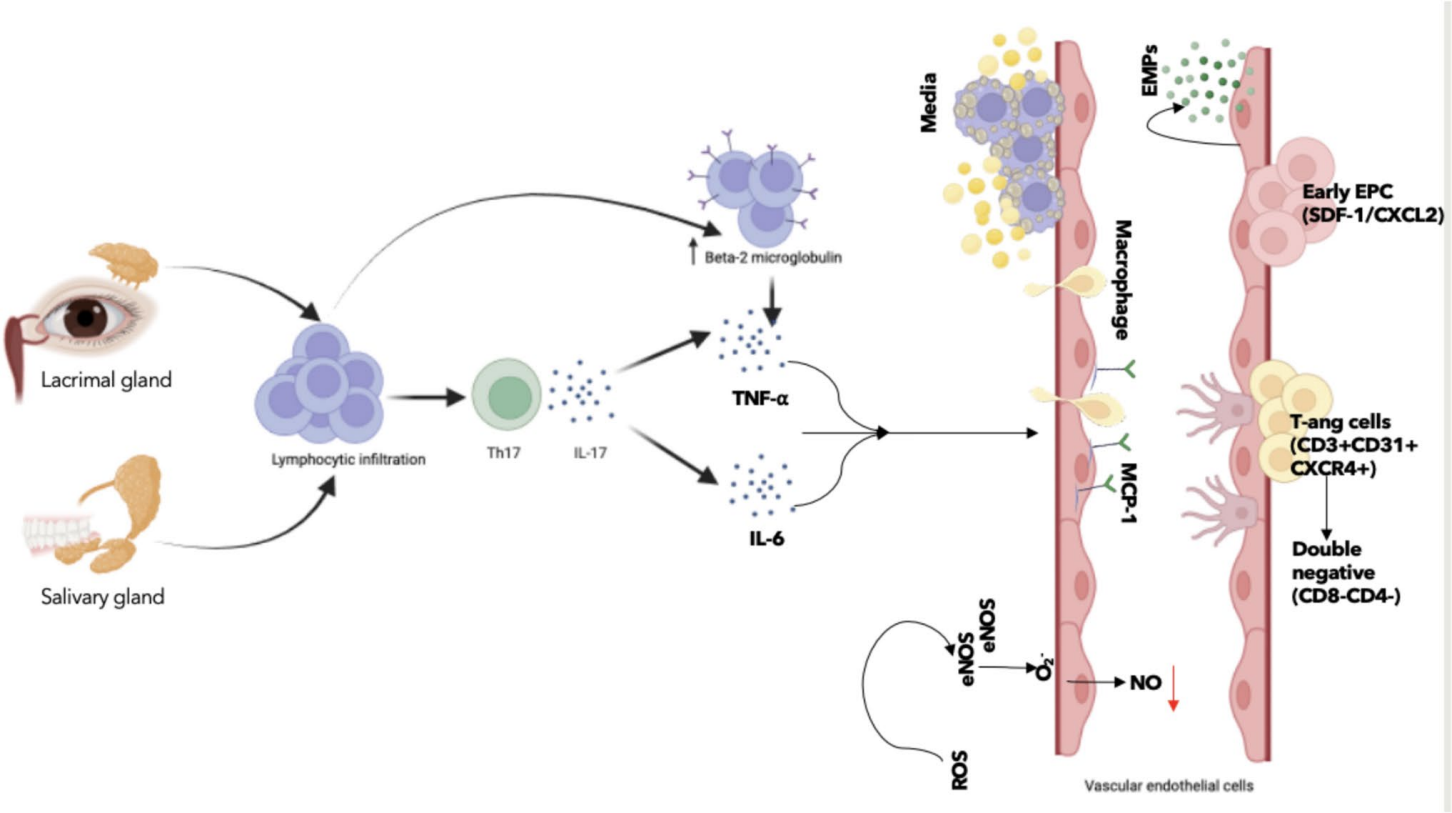
Glandular inflammation and vascular dysfunction in Sjögren’s syndrome. Lymphocytic infiltration of the lacrimal and salivary glands activates Th17 cells and promotes the release of IL- 17, TNF-α, and IL- 6. These cytokines induce macrophage activation, MCP- 1 expression, and endothelial injury through oxidative stress (ROS) and eNOS uncoupling, leading to reduced nitric oxide (NO) production. The vascular compartment shows endothelial microparticles (EMPs), extracellular vesicles (EVPs), and infiltration by specific immune cell populations, including early endothelial progenitor cells (EPCs, SDF- 1/CXCL2 +), T-angiogenic cells (CD3⁺CD31⁺CXCR4⁺), and double-negative T cells (CD8⁻CD4⁻), all contributing to vascular inflammation and dysfunction

The contribution of EPCs and their derivatives to ED in SS has also been investigated. Evidence shows an accumulation of senescent T-angiogenic (Tang) cells (CD3⁺CD31⁺CXCR4⁺) in pSS, correlating with ESSDAI scores, IL- 17 concentrations, and overexpression of stromal cell-derived factor- 1 (SDF- 1/CXCL12) in minor salivary glands [86]. These cells are known to promote cytotoxicity, pro-inflammatory activity, and pathological neoangiogenesis, all of which aggravate endothelial injury and enhance disease activity [86]. Figure 4 illustrates the interplay between glandular inflammation and vascular dysfunction in Sjögren’s Syndrome, highlighting how Th17-mediated cytokine release, EPC and T-angiogenic cell infiltration, and oxidative stress converge to impair endothelial integrity and promote disease activity.

Further supporting these findings, increased levels of endothelial microparticles (EMPs) and an altered early-to-mature EPC ratio (CD34⁺CD309⁺CD133⁺ vs. CD34⁺CD309⁺CD133⁻) have been observed in SS, indicating endothelial apoptosis and a reactive mobilization of progenitor cells from the bone marrow aimed at vascular repair [87].

Patients with pSS are at increased risk of cardiovascular disease, particularly subclinical atherosclerosis [79, 80, 88, 89]. Meta-analyses have shown elevated carotid intima-media thickness (CIMT) and pulse wave velocity (PWV) in pSS cohorts. These changes are driven by chronic inflammation, decreased levels of cardioprotective hormones—especially among postmenopausal women—sedentary lifestyle, and persistent endothelial dysfunction [88, 89]. Neurological manifestations such as migraine, cognitive disturbances, and Raynaud’s phenomenon have also been associated with the presence of anti-SSA antibodies and ED-related neurovascular impairment [90].

## Systemic sclerosis and endothelial dysfunction

SSc is a chronic autoimmune connective tissue disease characterized by cutaneous and visceral fibrosis, affecting the skin, lungs, kidneys, gastrointestinal tract, and cardiovascular system [91, 92]. Based on the extent of fibrosis and autoantibody profiles, SSc is classified into limited cutaneous (lcSSc) and diffuse cutaneous (dcSSc) subtypes. Central to its pathogenesis is vascular remodeling driven by endothelial injury, positioning ED as a key mechanistic contributor [38, 92].

SSc patients exhibit pronounced vascular abnormalities, including microvascular injury, blunted post-occlusive hyperemia, capillary loss, and digital ulcers (DUs), attributable to ED-impaired vasodilation and irregular arterial networks [93–95] DUs, often among the earliest complications, are associated with reduced FMD and microvascular dysregulation [94, 96, 97]. Arterial stiffness (AS), diminished arterial compliance, and elevated levels of biomarkers such as endothelial microparticles (EMPs; CD31⁺/CD42b⁻), asymmetric dimethylarginine (ADMA), and symmetric dimethylarginine (SDMA) further reflect ED severity, particularly in lcSSc, implicating NOS inhibition and inflammatory pathways [95, 98–100].

Chronic ED exacerbates cardiovascular risk in SSc. Elevated ADMA levels promote macrovascular injury and subclinical atherosclerosis, evidenced by increased CIMT and atheromatous plaque (AP) formation [38, 99, 101, 102]. Inflammation-mediated vascular remodeling—correlated with thrombomodulin, CRP, IL- 6, and CIMT—contributes to cardiac hypertrophy through increased afterload [92]. Additionally, glucocorticoid therapy may further accelerate atherosclerosis by inducing dyslipoproteinemia [102].

ED also plays a fundamental role in multi-organ complications. Skin involvement, indicated by elevated von Willebrand factor antigen (vWF-Ag) and higher modified Rodnan Skin Scores (mRSS), reflects ongoing endothelial injury, thrombosis, and defective angiogenesis [103, 104]. Pulmonary arterial hypertension (PAH) arises from arterial stiffness and microvascular damage, while renal impairment correlates with increased SDMA and decreased estimated glomerular filtration rate (eGFR) [95, 97]. Gastrointestinal and periodontal involvement further underscore the systemic nature of ED in SSc [105, 106].

**Fig. 5:**
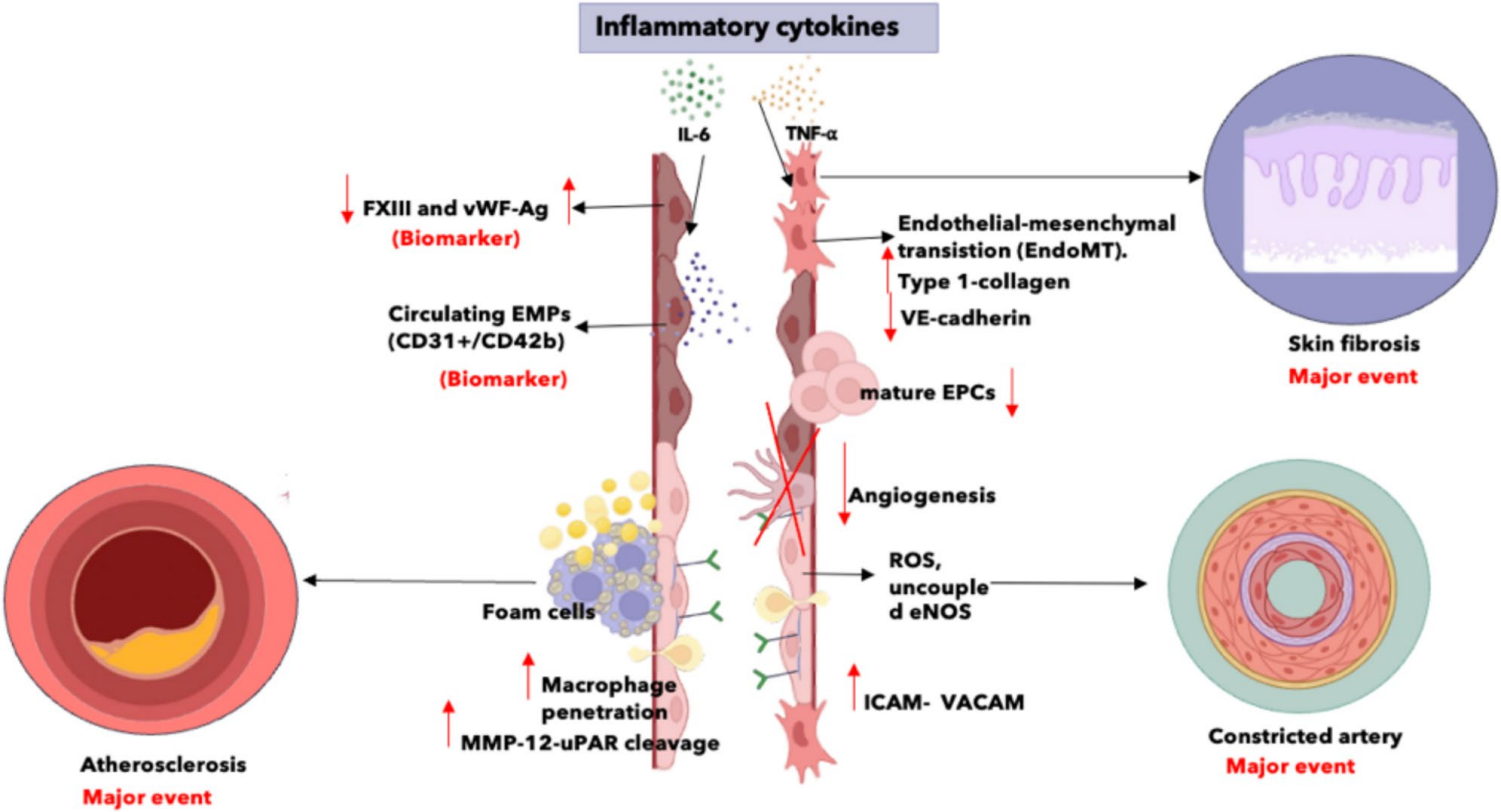
Inflammatory and fibrotic pathways in systemic sclerosis. Increased macrophage infiltration and foam cell formation enhance atherosclerosis, while endothelial-mesenchymal transition (EndoMT)—characterized by reduced VE-cadherin and increased type- 1 collagen—promotes skin fibrosis. Concurrently, elevated reactive oxygen species (ROS) production and uncoupled endothelial nitric oxide synthase (eNOS) induce arterial constriction. Biomarkers such as reduced FXIII and vWF-Ag, increased circulating endothelial microparticles (EMPs, CD31⁺/CD42b), and enhanced MMP- 12-uPAR cleavage highlight these endothelial disturbances

Endothelial-to-mesenchymal transition (EndoMT) has emerged as a critical pathway in SSc, driving fibrosis via activation of fibroblasts in dermal, pulmonary, and renal vasculature. This process is marked by loss of endothelial markers (e.g., CD31, VE-cadherin), acquisition of mesenchymal markers (e.g., α-SMA, collagen I), and glycolytic disturbances such as lactate accumulation, which impair angiogenesis and promote myofibroblast differentiation [107, 108]. NADPH oxidase-derived ROS and circulating microRNAs further contribute to EndoMT and fibrotic progression [109]. Figure 5 illustrates the central role of EndoMT in SSc, depicting how endothelial injury, oxidative stress, and immune-mediated signaling converge to promote fibroblast activation, vascular remodeling, and tissue fibrosis.

Therapeutic approaches targeting ED in SSc include calcium channel blockers, phosphodiesterase- 5 inhibitors, and endothelin- 1 antagonists, all aimed at improving vasodilation. Statins have been shown to enhance endothelial progenitor cell (EPC) function [110]. Emerging treatments, such as tetrahydrobiopterin (BH4), increase nitric oxide (NO) bioavailability, improving FMD and promoting DU healing without exacerbating oxidative stress [111]. Iloprost, a prostacyclin analog, reduces oxidative stress and collagen synthesis in pulmonary microvascular endothelial cells [112]. However, combination therapies (vasodilators + immunosuppressants) yield mixed outcomes, suppressing TGF-β and IL- 6 but failing to normalize FMD or angiogenesis markers (VCAM, ICAM, VEGF), underscoring SSc’s multifactorial complexity [113].

## Polymyalgia rheumatica and endothelial dysfunction

**PMR** is one of the most prevalent inflammatory rheumatic disorders in older adults, typically affecting individuals over the age of 50. It is characterized by chronic musculoskeletal pain and stiffness, particularly around the shoulders and hips, and is associated with elevated levels of acute-phase reactants such as CRP and erythrocyte sedimentation rate (ESR) [114, 115]. ED has been documented in patients with PMR, potentially arising from a combination of inflammatory processes and genetic predisposition [116]. In one study, patients with PMR showed significantly reduced flow-mediated dilation (FMD), along with elevated CRP and ESR levels compared to controls [115]. Notably, after one year of corticosteroid therapy, a substantial improvement in FMD was observed, although values remained below those of the control group. These findings suggest that, while steroidal treatment can attenuate systemic inflammation and partially restore endothelial function, it may not completely reverse vascular injury [115].

Genetic factors potentially contributing to ED in PMR have also been explored. A substitution mutation (786 T > C) in the NOS3 gene, encoding endothelial nitric oxide synthase (eNOS), was identified at a higher frequency in PMR patients and was strongly associated with increased ESR levels [116]. This homozygous C variant was found to impair nitric oxide (NO) bioavailability, reducing vasodilation and promoting endothelial vulnerability, thereby potentially exacerbating vascular dysfunction in PMR.

The pathogenesis of PMR appears to involve a complex interaction between ED and immune dysregulation. Components of both innate and adaptive immunity have been implicated. Increased levels of EMPs and reduced numbers of EPCs are thought to contribute to innate immune activation, while heightened CD8⁺ T-cell activity may stimulate endothelial cells and enhance adaptive immune responses [114]. These mechanisms collectively promote persistent vascular inflammation and may play a central role in disease progression.

## Conclusions

The connection between RDs and ED is complex and influenced by various factors such as chronic inflammation and immune system dysregulation. This review highlights that people with RDs, OA**,** RA, SLE, AS, PsA, SS, SSc, and PMR are at a higher risk of developing ED. ED significantly contributes to CVD by impacting NO production, increasing oxidative stress, and promoting pro-inflammatory and prothrombotic activities. Evidence suggests that patients with RDs, even without traditional cardiovascular risk factors, exhibit a significant connection between RD-related inflammation and ED. Additionally, ED plays a crucial role in the initiation and progression of atherosclerosis, thus elevating the cardiovascular risk in these individuals. This review has compiled and synthesized current research to provide a comprehensive understanding of the underlying molecular mechanisms linking major RDs and ED. The findings emphasize the importance of acknowledging ED as a significant factor in the development of cardiovascular complications in patients with RDs. It also identifies potential biomarkers and therapeutic targets for reducing cardiovascular risk in this population.

### Future perspectives

Future research should aim to clarify the specific molecular mechanisms underpinning the bidirectional relationship between ED and RDs. Longitudinal studies and robust clinical trials are essential for validating current findings and developing effective interventions. Investigating novel biomarkers for early ED prediction could significantly enhance diagnosis and treatment strategies. Priority should be given to assessing therapies targeting inflammatory pathways and endothelial function, including anti-TNF agents, statins, and vitamin D supplementation. Exploring genetic and environmental contributors to ED within different RD populations could inform personalized treatments and improve clinical outcomes. Indeed, emerging research on circular RNAs suggest their promising roles as biomarkers and therapeutic targets for modulating endothelial function and inflammation in RDs opening avenues for precision medicine [117]. Overall, deepening our understanding of the interplay between RDs and ED will facilitate innovative preventive and therapeutic approaches, ultimately improving patient quality of life and reducing cardiovascular morbidity and mortality.

## Data Availability

No datasets were generated or analysed during the current study.
